# Nanosecond pulsed electrical fields enhance product recovery in plant cell fermentation

**DOI:** 10.1007/s00709-020-01534-9

**Published:** 2020-07-10

**Authors:** Fatemeh Rajabi, Christian Gusbeth, Wolfgang Frey, Jan Maisch, Peter Nick

**Affiliations:** 1 Molecular Cell Biology, Botanical Institute, Karlsruhe Institute of Technology, Karlsruhe, Germany; 2 Institute for Pulsed Power and Microwave Technology (IHM), Karlsruhe Institute of Technology, Karlsruhe, Germany

**Keywords:** Tobacco (*Nicotiana tabacum* L.), Plant cell fermentation, Nanosecond pulsed electric fields, Reversible electroporation, Metabolite recovery, Nicotine

## Abstract

The potential of pharmacologically active secondary plant metabolites is limited by the low yield from often rare plants, and the lack of economically feasible chemical synthesis of these complex compounds. Plant cell fermentation offers an alternative strategy to overcome these constraints. However, the efficiency of this approach is limited by intracellular sequestration of the products, such that continuous bioprocessing is not possible. As a precondition for such a, more attractive, continuous process, it is of great importance to stimulate the export of the product into the medium without impairing viability and, thus, the productivity of the cells. Using nicotine alkaloids of tobacco as a case study, an alternative strategy is explored, where nanosecond pulsed electric fields (nsPEFs) are applied for the efficient downstream recovery of the products. To maintain cell viability and allow for the further use of biomass, cells were exposed to strong (1–20 kV·cm^−1^), but very short (10–100 ns) electric pulses, which leads to a temporary permeabilisation of cell membranes. Using two transgenic cell lines, where two key genes involved in the metabolism of the anti-Alzheimer compound nornicotine were overexpressed, we could show that this nsPEF treatment improved the partitioning of some nicotine alkaloids to the culture medium without impairing viability, nor the synthesis of alkaloids. However, this release was only partial and did not work for nornicotine. Thus, nsPEFs produced a fractionation of alkaloids. We explain this electrofractionation by a working model considering the differential intracellular compartmentalization of nicotineic alkaloids.

**Electronic supplementary material:**

The online version of this article (10.1007/s00709-020-01534-9) contains supplementary material, which is available to authorized users.

## Introduction

Plants are endowed with an impressive ability to produce metabolites, especially secondary compounds (Goossens et al. 2003; Anarat-Cappillino and Sattely 2014). This metabolic proficiency has been explored and exploited by humans ever since, especially for medicinal applications. In our time, the increasing popularity of phytomedicinal products has spawned a corresponding increase in the global demand for natural compounds. As a result, pharmaceutically important plants are increasingly exploited on an industrial scale—a process which, if allowed to go on unchecked, may push many of these, often rare, plants to the verge of extinction. For instance, the bark of up to twelve individual *Taxus brevifolia* trees was needed to provide the taxol required to cure one ovarian cancer patient (Joyce 1993). Since this approach is not sustainable, parallel efforts have been launched to find alternative ways for the production of these compounds, rather than relying solely on natural sources (Bourgaud et al. 2001). Plant cell fermentation represents a promising alternative and has been successfully established on an industrial scale for taxol (reviewed in Malik et al. 2011; Imseng et al. 2014). However, the number of cases, where plant cell fermentation is used, remains fairly limited. One of the major limitations is the fact that most of the valuable metabolites tend to remain intracellular and are not secreted to the medium, such that the biomass has to be harvested and extracted in batch, requiring that a new production cycle has to be initiated de novo (Cai et al. 2012). A system, where a compound of interest would leave the cell without impairing viability and, thus, productivity, would allow a continuous process, such that production would become easier and more cost-efficient. Furthermore, continuous release would prevent that these metabolites become sequestered in inner organelles, such as vacuoles, which would significantly decrease product inhibition and therefore improve productivity (Chattopadhyay et al. 2002). Secretion of valuable metabolites to the culture medium can significantly decrease downstream processing costs (Wilson and Roberts 2012), but is often limited by the pecto-cellulosic cell wall, and more importantly, by the plasma membrane, sometimes also by the membranes of intracellular compartments (Georgiev et al. 2009).

In cases, where no natural secretion of secondary compounds, either through membrane transporters or through exocytosis, is available, technical approaches have been employed to induce the release of the metabolites into the medium, such as permeabilisation using pH shock (Thimmaraju et al. 2003), chemical treatments or electroporation (Galindo, 2016). As a merely physical approach, electroporation has found intensive application in food science, because it does not require the addition of potentially hazardous and sometimes costly chemicals. Irreversible permeabilisation of the plasma membrane allows to increase extraction yields of biomolecules (such as colourants and antioxidants), for instance, during the production of fruit juices (Galindo, 2016). In classical electroporation, long pulses (in the range of micro-, or even milliseconds), but at less intense electric field strength, will produce hydrophilic pores in the plasma membrane (Angersbach et al. 2000). Due to the charge, the polar head of phospholipids will tilt the entire phospholipid, resulting in an increase of cell membrane permeability (Neumann and Rosenheck 1972; Chopinet and Rols 2014). In fact, electroporation has been used to release betacyanin from suspension cultures of *Chenopodium rubrum*, or berberine from suspension cultures of *Thalietrum rugosum*. However, electroporation, while efficiently releasing desired secondary products from the cells, is causing irreversible damage, such that continuous processes become impossible, necessitating batch extraction (Brodelius et al. 1988).

Thus, the need for cell permeabilisation collides with the need to preserve cell viability and proliferation at the same time, limiting the applicability of electroporation as a method for compound release (Brodelius 1988; Park and Martinez 1992). Treatment of cells at very high intensity, but much shorter duration (in the ns-range), allows to resolve this dilemma, because the field can penetrate into the cell interior before the plasma membrane is fully charged (Frey et al. 2006; Eing et al. 2009; White et al. 2011). It is possible, though, to permeabilise also the plasma membrane in a transient manner, by tuning the physical parameters of the nanosecond Pulsed Electrical Field (nsPEF) treatment (Brodelius 1988; Hapala 1997; Knorr and Angersbach 1998). By adjusting the number and the energy of pulsing, it is even possible to achieve a reversible permeabilisation of the plasma membrane, such that the cells remain viable and show often specific long-term responses to the treatment, such as remodelling of the cytoskeleton (Berghöfer et al. 2009; Hohenberger et al. 2011), changes of nuclear positioning and elongation axis (Kühn et al. 2013) or developmental responses, such as the formation of a palmella (Bai et al. 2017).

In the current work, we explored the use of nsPEFs for plant cell fermentation of a pharmaceutically interesting compound, nornicotine, using metabolic engineering in transgenic strains of BY-2 (*Nicotiana tabacum* L. cv Bright Yellow 2), a model cell line for plant research (Nagata et al. 1992). Nornicotine has acquired considerable interest, because it interferes with the amyloid β-protein, responsible for the progression of Alzheimer disease (Dickerson and Janda 2003). Although derived from tobacco, this line does not accumulate nicotine under standard conditions, but can be induced to do so after elicitation by jasmonic acid (Rajabi et al. 2017). By overexpression of the key enzyme *Ntab*MPO1, the accumulation of nicotine can be strongly promoted. Since no nornicotine was detected, we engineered a different strain, where a nicotine demethylase from a nornicotine producing tobacco species (*N. tomentosiformis*), *NtomCYP82E4*, was overexpressed. However, still, we were not able to recover significant amounts of nornicotine. However, when the *NtomCYP82E4* overexpressor line was incubated with conditioned medium from *Ntab*MPO1 cells, high levels of nornicotine were obtained that were equivalent to those levels found in the leaves of the converter species *N. tomentosiformis*. While a part of the nicotine was released to the medium, probably by the activity of membrane transporters, the majority of the produced alkaloids, as often observed for secondary metabolites, remained sequestered inside of the cell (Rajabi et al. 2017). In the current study, we used this experimental model to assess, whether mild nsPEF treatments are able to induce a transient increase of membrane permeability and, thus, the release of alkaloids, while preserving cell viability and thus productivity of the cell culture.

## Material and methods

### Plant cell material, cell culture and elicitation

All work was done with tobacco suspension cells (*Nicotiana tabacum* L. cv Bright Yellow 2 (Nagata et al. 1992) was cultivated in liquid medium containing 4.3 g l^−1^ Murashige and Skoog salts (Duchefa, http://www.duchefa.com), 30 g l^−1^ sucrose, 200 mg l^−1^ KH_2_PO_4_, 100 mg·l^−1^ inositol, 1 mg l^−1^ thiamine and 0.2 mg l^−1^ (0.9 μM) 2,4-D, pH 5.8. The cells were subcultivated weekly, inoculating 1.0–1.5 ml of stationary cells into fresh medium (30 ml) in 100 ml Erlenmeyer flasks, at 26 °C under constant shaking on a KS260 basic orbital shaker (IKA Labortechnik, http://www.ika.de) at 150 rpm. Every 3 weeks, stock calli were subcultured on media solidified with 0.8% (*w*/*v*) agar (Roth, http://www.carlroth.com). Cloning, transformation and establishment of stable transgenic tobacco BY-2 cells were conducted as described previously (Rajabi et al. 2017).

Briefly, stable cell lines overexpressing *NtabMPO1* and *NtomCYP82E4* were obtained through a *Agrobacterium*-based transformation method (Buschmann et al. 2011) with minor modifications (Rajabi et al. 2017). Suspension cultures and calli of the transgenic strains *NtabMPO1*ox and *NtomCYP82E4*ox were cultivated in the same media as non-transformed wild-type cultures (BY-2 WT), but supplemented with 50 mg·l^−1^ kanamycin. Jasmonic acid (OlChemIm, http://www.olchemim.cz/), dissolved in ethanol, was used as an elicitor to increase the production of our target compounds and was added to the culture medium to a final concentration of 100 μM. The addition of an equivalent volume of ethanol alone served as solvent control. If not stated otherwise, the samples for nsPEF treatment and alkaloid analysis were collected at the peak of mitotic activity, 3 days after subcultivation. Each experiment was accompanied by a mock control, where the cells were passing the device, just omitting the pulsing.

### Alkaloid extraction

Nicotine alkaloids were extracted according to Häkkinen et al. (2004) with the following modifications: To extract tobacco alkaloids, 1 g fresh weight of BY-2 cells was dispersed in 2 ml of water. The mixture was basified with 3 ml of 3.3% NH_4_OH. To release the cell content, cells were lysed by ultrasonication for 2 min by means of a high-efficiency ultrasound device (UP 100H, Hielscher, https://www.hielscher.com), pulsed with 0.5 s intervals using an amplitude of 100%. The lysate was spun down for 15 min at 2100 ×*g* (Z 383 K, Hermle KG, https://www.hermle.de), and the supernatant was collected and extracted with 10 ml dichloromethane. The mixture was incubated for 30 min at 20 °C on an orbital shaker (150 rpm). Subsequently, the polar dichloromethane layer was separated and collected through a 50-ml separation funnel. In order to improve the efficiency of extraction, this step was repeated. Precipitated proteins were separated by centrifugation from the collected polar phase at 2100 ×*g* for 15 min. In the next step, the clear lower phase was concentrated in a rotary evaporator (Büchi® R-205, http://www.buchi.com) under a reduced pressure of 550 millibars and a temperature of 40 °C. After complete evaporation of dichloromethane, the extract was dissolved in 500 μl of 80% (*v*/v) methanol for HPLC analysis.

### Separation of alkaloids by high-performance liquid chromatography

The Agilent-1200-Series HPLC system equipped with a diode array detector (G1315D), the Agilent ChemStation software and a Phenomenex Gemini-NX 5 μ C18 110A 150 mm × 4.6 mm column (Phenomenex, http://www.phenomenex.com/) was used at a column temperature of 35 °C and a flow rate of 1.0 ml·min^−1^. The injection volume was 20 μl for cell extracts and 30 μl for medium extracts; peaks were quantified at 260 nm. UV spectra were collected over the wavelength range from 200 to 700 nm. Eluent A contained 10% acetonitrile in 20 mM ammonium formate, adjusted to pH 8.7, and eluent B consisted of 100% acetonitrile. A gradient program was employed, composed of a sequence of linear gradients with an initial step of 100% A to 80% A and 20% B over the first 10 min, followed by a second gradient to 10% A and 90% B over the next 10 min, and a final step to 100% B from 21 min after injection till the end of the run at 30 min after injection (Trehy et al. 2011). The reference alkaloids nicotine and nornicotine were purchased from Sigma-Aldrich (Munich, Germany) and used for sample spiking to verify the identified peaks (Online Resource 1). Data represent mean values and standard errors from three independent experimental series. The significance of the observed differences was probed with the non-parametrical Friedman test.

### Viability assay

Mortality in response to nsPEF treatment was measured by the Evans Blue Dye Exclusion test (Gaff and Okong’o-Ogola 1971). Aliquots of 500 μl of pulsed cell suspension were stained with 2.5% (*w*/*v*) Evans Blue solution (Sigma-Aldrich) for 5 min. Afterwards, the dye was drained using custom-made chambers (Nick et al. 2000), and cells were washed three times, each for 5 min with fresh MS medium and the frequency of the unstained (viable) cells was determined under bright-field illumination using an AxioImager Z1 microscope (https://www.zeiss.de). For each individual sample, at least 1000 cells were scored. Data represent mean values from three biological replicates.

### Basic experimental setup for nsPEF treatment

**Fig. 1:**
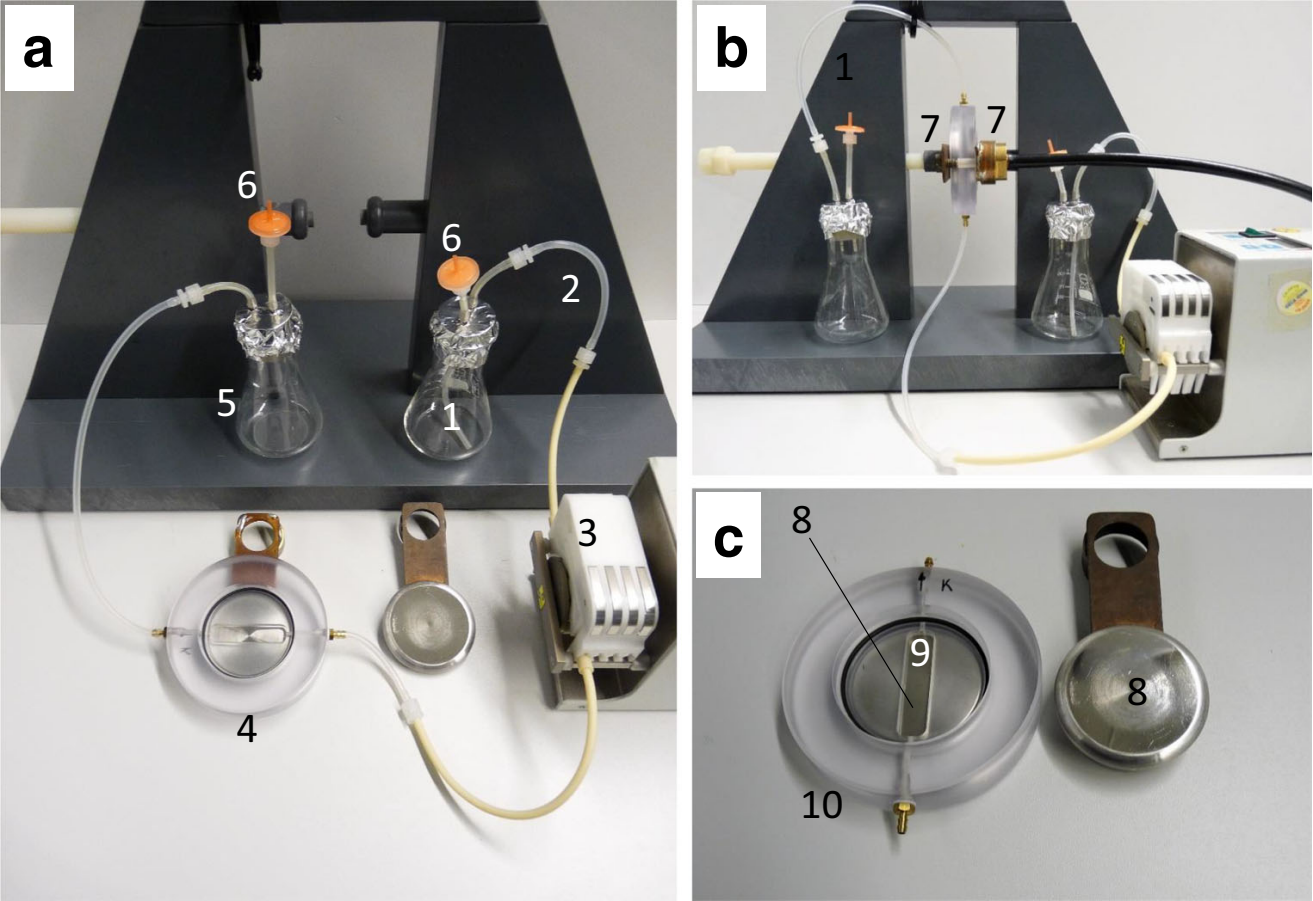
Experimental setup for continuous-flow nsPEF treatment of tobacco BY-2 cells. **a** Overall setup with dismantled pulsing chamber. 1 donor flask, 2 silicon tubing 3 peristaltic pump 4 pulsing chamber (dismantled to show the setup) 5 recipient flask, 6 ventilation ports with microfilters to safeguard sterility. **b** Device mounted ready to use. 7 High-voltage connectors. **c** Details of the dismantled pulsing chamber. 8 electrodes 9 pulsing gap (4 mm width, volume 2 ml) 10 polycarbonate housing. Note that upon mounting, the two electrodes are spatially separated by the polycarbonate spacer such that the field is acting across the gap (in z-direction from the observer).

The BY-2 cell suspensions were treated in a continuous flow treatment chamber, manufactured at the Institute for Pulsed Power and Microwave Technology (Karlsruhe Institute of Technology, Germany) (Fig. 1). The details of the pulsed generator system have been reported previously by Eing et al. (2009) and Goettel et al. (2013): The custom-made treatment chamber consisted of two stainless steel electrodes with a diameter of 60 mm, oriented in parallel and separated by a 4-mm gap integrated into a transparent polycarbonate housing (Fig. 1c). For each experiment, a volume of 30 ml of cells was continuously treated with nsPEF (Fig. 1b). The cell suspensions passed the treatment chamber vertically from the bottom via a sterilised silicone rubber tubing, driven by a peristaltic pump (Ismatec Ism 834C, Switzerland) at a constant flow rate of 5.3 ml·min^−1^. After passing the outlet at the top of the chamber, the suspension was collected in an empty, sterilised 100-ml Erlenmeyer flask sealed by a silicone plug (Fig. 1a). In this work, the transmission line pulse generator delivered rectangular pulses with a voltage amplitude of 2 kV, corresponding to an electric field strength of 5 kV·cm^−1^. The distance between the electrodes was 4 mm and the treatment volume was 2 ml.

### Experimental setup and nsPEF parameters

To investigate whether nsPEF treatment can stimulate the release of the desired alkaloids without affecting cell viability and cell growth, cells were pulsed with different, specific parameter sets, defined as pulsing indices (PI) 1–3 that were tested in preparatory experiments (Table 1). Based on the outcome of this preparatory study, PI3 was chosen as optimal parameter set. Pulsing index 0 represents a control treatment without any nsPEF application.

**Table 1:** Pulsing indices (PI) tested in preparatory experiments defining field strength, pulse duration, conductivity, flow rate, frequency, number of pulses and electric strength. These PI were tested using WT, *Ntab*MPO and *Ntom*CYP overexpressor BY-2 cell lines. For all further investigations, PI3 was selected

Pulsing index (PI)	Field strength (kV cm^−1^)	Pulse duration (ns)	Conductivity (mS cm^−1^)	Flow rate (ml s^−1^)	Frequency (Hz)	Number of pulses	Input specific energy (J g^−1^)	Cell mortality
0	-	-	-	-	-	-	-	< 10%
1	5	25	4	0.0892	2	45	0.112	~ 30%
2	20	50	5.95	0.0892	0.25	6	0.667	~ 50%
3	5	100	4	0.0892	0.5	11	0.112	< 20%

When many samples had to be treated, the experiment was repeated sequentially, and the cells were kept on an orbital shaker within an incubator at 25 °C in dark at 150 rpm prior to sample preparation to ensure that potential effects of environmental factors were as constant as possible. Both short-term (2 h after the nsPEF treatment) and long-term (24 h after the nsPEF treatment) responses were investigated. To follow the short term responses, the suspensions were pulsed at day 3 after subcultivation, and cell viability, intracellular and secreted alkaloids were measured 2 h after the pulse treatment. To observe the long-term responses to nsPEF treatment, the pulsed samples were returned to the incubator and further cultivation under standard conditions (25 °C, dark, shaking at 150 rpm) until 24 h after the nsPEF treatment, when viability and alkaloid contents were quantified. As a negative control, the suspension was cultivated and treated in the same device in a mock experiment, just omitting the application of nsPEF.

## Results

### Adjusting parameters for non-lethal nsPEF treatment

The pulse conditions for electropermeabilisation of BY-2 had to be adjusted such that on the one hand, a permeabilisation of the membrane would be achieved, while, on the other hand, the cells should remain viable and restore proliferation. A cell line with reversible permeabilisation of the membrane would be able to regenerate by cell division within a few days, while cells with irreversible damages would face cell death. The critical factors determining the intensity of damage are field strength, pulse duration and number of pulses (Kühn et al. 2013). In fact, these three factors are the main determinants of the total energy level exposed to the cells. For this reason, a set of different pulsing indices were tested for their ability to release nicotineic alkaloids into the medium, while preserving viability (Table 1). According to our results, pulsing index 3 (field strength 5 kV cm^−1^, pulse duration 100 ns, 11 pulses, yielding 0.112 J g^−1^, details see Table 1) was balancing these requirements in an optimal manner, such that the subsequent experiments were conducted with these parameters.

### Effect of nsPEFs on alkaloid release

In our previous work (Rajabi et al. 2017), we had observed that cells overexpressing *NtomCYP82E4* were able to accumulate high amounts of nornicotine, if they were confronted with the culture filtrate from cells overexpressing *NtabMPO1*. This could, alternatively, also be achieved by simply co-cultivating the two cell lines, which was the strategy chosen for nsPEF treatment in the current work. Briefly, equal volumes of the *NtabMPO1* overexpressor, and *NtomCYP82E4* overexpressors were cultivated in the double volume of added fresh medium, and elicited with jasmonic acid at a final concentration of 100 μM. Three days after cultivation, the cells were pulsed with pulsing index 3 (11 pulses at a frequency of 4 Hz and a pulse duration of 100 ns, Table 1). A parallel mock control as an independent entity comprised cells that were passing the same device, just with the voltage switched off.

**Fig. 2:**
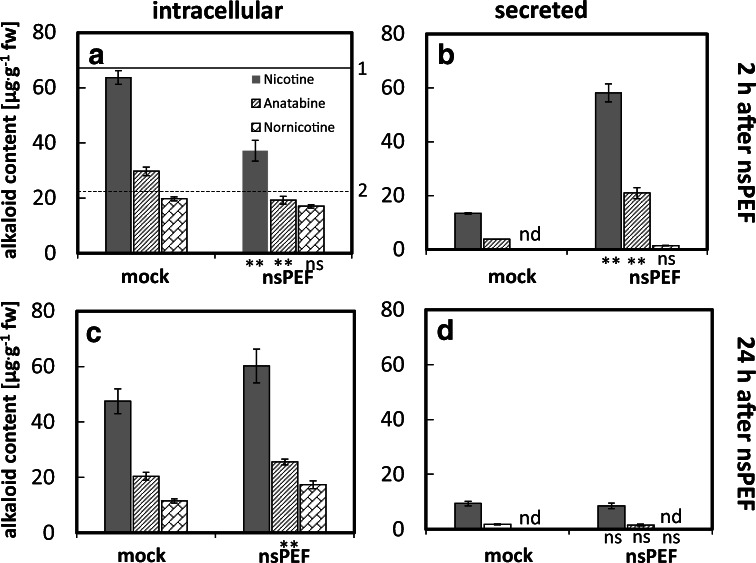
Alkaloid content of cells overexpressing *NtomCYP82E4* co-cultivated together with *NtabMPO1* (to achieve accumulation of nornicotine) 2 h (**a**, **b**), or 24 h (**c**, **d**) after nsPEF treatment either accumulated inside of cells (**a**, **c**), or secreted into the medium (**b**, **d**). Cells were either pulsed with nsPEFs (100 pulses, 5 kV cm^−1^), or subjected to a mock treatment using the same device, just omitting the pulsing. Levels of nornicotine below detection limit are indicated as non-detectable (nd). 1 (solid line, nicotine) and 2 (dashed line, nornicotine) represent the resting levels without any treatment (prior-pulsing control) measured under these conditions. Data represent means and standard errors from three independent experimental series. Significant differences to the non-treated cells (control) were tested by the non-parametrical Friedman test and are indicated by *(*P* < 0.05) or **(*P* < 0.01), respectively.

Alkaloid contents in the medium, determined immediately after nsPEF treatment (Fig. 2a), revealed a strong (around 5-fold) increase in the level of nicotine and anatabine in the culture medium, concomitant with a decrease of relative intracellular alkaloids. In contrast, nornicotine, although clearly induced in the cell interior, was barely detectable in the culture medium. These results are consistent with a working model, where pulsing at these parameters was successful in releasing nicotine and anatabine from the cell into the medium, while this was not efficient in the case of nornicotine (Fig. 2b). A comparison with intracellular levels of nicotine and nornicotine in cells prior to the treatment (Fig. 2a, solid and dashed lines) showed that the mock treatment did not alter alkaloid accumulation.

**Fig. 3:**
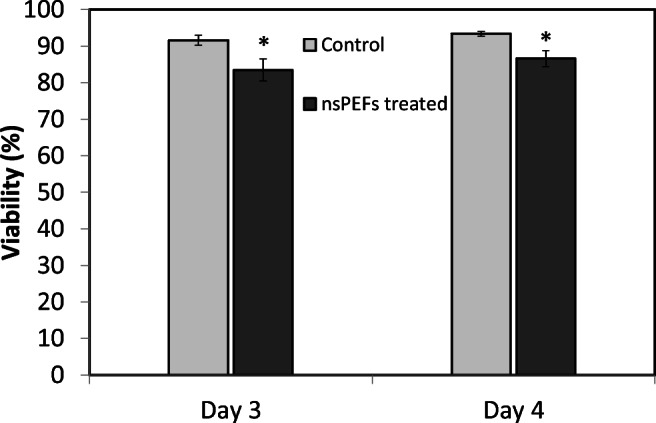
Viability of nsPEF treated cells compared with untreated cells (control) immediately and 24 h after treatment (*n* = 1000). Cells were subjected to nsPEFs at day 3 after subcultivation. Data represent mean and SE from three independent experimental series. Significant differences to the non-treated cells (control) assessed by a Student’s *t* test are indicated by *(*P* < 0.05).

When cell mortality was quantified immediately after the nsPEF application, it was seen to be reduced only slightly (by 8%) compared with the control (Fig. 3). This very modest increase in mortality cannot account for the observed 5-fold enhancement of alkaloid secretion following nsPEF treatment, which means that this increase cannot be due to cell death.

To test, whether the intracellular alkaloid pool would recover in the time after the pulse treatment, intracellular levels of nicotine, anatabine and nornicotine were measured also at 24 h after nsPEF treatment (Fig. 2c, d). Interestingly, the intracellular content of nicotine not only recovered to the level observed before the pulse but also was overshooting beyond that seen in the control (which had dropped during this time interval) (Fig. 2c). For anatabine, a similar recovery was seen, however, without this overshooting effect. For nornicotine, where the nsPEF treatment had not caused any depletion of the intracellular pool, there was also no long-term change in intracellular abundance over the 24 h that followed the nsPEF treatment. For none of the alkaloids, the increase observed in the culture medium immediately after the pulse was seen 24 h later (Fig. 2d), indicative of a situation, where the permeabilisation did not persist for a long time. Consistent with such a transient effect, there were also no significant reductions in viability after 24 h (Fig. 3, day 4), which implies that any damage which might have been caused by the selected pulse index (PI3) had been fully compensated.

## Discussion

The current work was motivated by the idea to use nsPEFs for mild and reversible permeabilisation during plant cell fermentation in preparation of a continuous process flow as a more convenient alternative to batch extraction. We applied this approach to a modular design, where two engineered strains of the model cell culture tobacco BY-2 were co-cultivated that express two key enzymes in the synthetic pathway culminating in the formation of the anti-Alzheimer compound nornicotine (Rajabi et al. 2017). We could show that a mild permeabilisation by nsPEFs was able to stimulate the release of the alkaloids nicotine and anatabine without impairing the viability of the cellular system. We observed that around a third of the intracellular pool for these alkaloids was released. We also could show that nornicotine accumulated inside of the cell under these conditions. However, this accumulated nornicotine was not released by the treatment—in contrast to nicotine and anatabine. In principle, the increase of alkaloids in the medium might be caused nonspecifically, by cell death caused by the pulsing. Two arguments speak against this possibility. First, the mortality after pulsing was very low (less than 10%), which cannot account for the 5-fold increase of alkaloid levels in the medium. Second, dead cells should also release nornicotine. Thus, the alkaloid levels found in the medium must result from a specific process. We further found that the reduction of the intracellular pools of nicotine and anatabine in response to nsPEFs was fully compensated within the subsequent 24 h. Thus, we see, at several levels, a fractionation of alkaloid release: (i) at the level of the alkaloid type—nicotine and anatabine are released by nsPEFs, nornicotine is not, and (ii) at the level of intracellular pools: a part of nicotine and anatabine (around 1/3) is released, a larger part is not. In the following, we will discuss potential reasons for the compensation phenomenon, and especially the potential mechanisms behind this fractionation on the base of what is known about nicotine compartmentalisation and transport. It should be stated openly that at current, this can only be a working model, whose main purpose is to structure future research.

A possible reason for the recovery of intracellular alkaloid levels following the nsPEF-induced reduction nicotine and anatabine pools during the subsequent 24 h might be that nsPEF treatment could work as an external stimulus to activate alkaloid synthesis. Nicotine alkaloids are usually synthetised in the tobacco root and translocated to the leaves as a defence response to herbivores (Baldwin 2001). The temporary disruption of membrane integrity by nsPEF might therefore act as a stimulus signalling an herbivore attack, and, thus, induce synthesis of alkaloid synthesis genes. Previous work has already shown that nsPEF treatment can activate calcium influx (Flickinger et al. 2010), as well as the NADPH oxidase Respiratory burst oxidase Homologue (Bai et al. 2018), two of the major inputs for plant-stress signalling (reviewed in Riemann et al. 2015). The activation of these stress inputs might then be responsible for the synthesis of nicotine alkaloids as phytoalexins. A similar effect of PEFs has already been described earlier by Ye et al. (2004), where PEF treatment could induce oxidative burst and accumulation of secondary metabolites in suspension cultures of *Taxus chinensis*. However, it is also conceivable that it is the depletion of the internal nicotine pool that activates the expression of MPO1 as a limiting step of nicotine synthesis, followed by the recovery of the depleted pool. To test this, expression studies of MPO1 after modulation (and quantification) of the intracellular nicotine pool would be important.

There is, however, a much simpler model that can explain the recovery of the alkaloid pool, the alkaloids released in response to nsPEF treatment, might be simply recycled with the help of nicotine importers located in the plasma membrane, such as the permease NUP1 (Kato et al. 2015). This would also explain why the alkaloid levels in the medium during the 24 h following the pulse return to the initial value prior to the pulse.

**Fig. 4:**
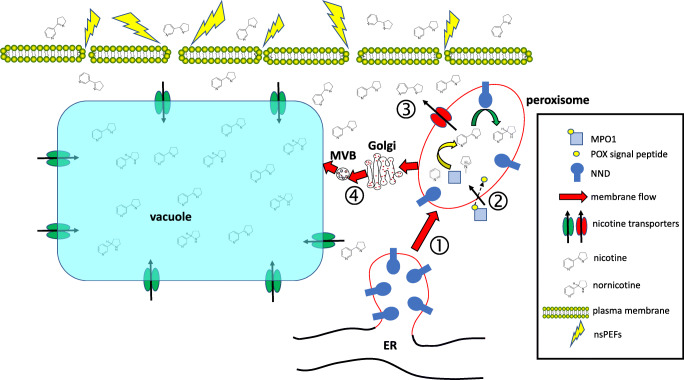
Working model to explain alkaloid fractionation by nsPEFs. The nicotine *N*-demethylase (NND) is anchored in the ER membrane by virtue of a *N*-terminal transmembrane domain and reaches the peroxisome by membrane flow ①, while *N*-methylputrescine oxidase 1 (MPO1) is imported into the peroxisomal lumen by virtue of a peroxisomal (POX) signal peptide ②. The fusion of 2,5-dihydropyridine and the *N*-methyl-Δ^1^-pyrrolinium cation into nicotine is catalysed by MPO1 in the peroxisomal lumen. A part of nicotine is exported into the cytoplasm ③ by an unknown transporter (red), while a part is further converted by the NND into nornicotine, which remains sequestered in the peroxisome, from where nornicotine, and the fraction of nicotine that had neither been exported into the cyotoplasm nor converted to nornicotine can reach the vacuole by membrane flow ④ via Golgi apparatus and Multivesicular Body (MVB). The cytoplasmic nicotine can also reach the vacuole by membrane transporters such as NtJAT1 (green). Upon mild and transient permeabilisation of the plasma membrane by the nsPEFs used in this study, a fraction of the cytoplasmic nicotine can be released into the medium, while the fraction of nicotine and the entire pool of nornicotine, which are sequestered in internal compartments (peroxisome, Golgi, MVB, vacuole), are not released.

Central to the current work was the finding that the nsPEF treatment caused a fractionation of alkaloid release, both in type (nicotine and anatabine are released, while nornicotine is not) and in proportion (around 1/3 of nicotine and anatabine are released, 2/3 are not). While the intracellular compartmentalisation of nicotine and nornicotine has remained surprisingly elusive despite decades of research, it is possible to derive some conclusions based on the localisation pattern of biosynthetic enzymes and nicotine transporters (Fig. 4): Nicotine is exclusively produced in the root and has to leave the synthetising cell (mainly pericycle cells) to reach the leaves through the xylem, driven by transpiration (Baldwin 2001). In the leaves, nicotine is stored in the vacuole (Shitan et al. 2009). This transport route requires several membrane passages. Since the pK_s_ values of nicotine are 7.9 for the pyridine ring, and around 6 for the pyrrolidine ring (Beckett et al. 1972), this molecule is either mono-protonated (in the cytoplasm) or even di-protonated (in the apoplast or in the vacuole). Several multidrug and toxic compound extrusion (MATE) transporters can sequester nicotine into the vacuole (Shoji et al. 2009). Among those, NtJAT1 is interesting because of its differential localisation: it has been shown to sequester nicotine into the leaf vacuole (Morita et al. 2009), but in the root might act as nicotine exporter from cytoplasm to apoplast (Shitan et al. 2009). In both cases, this transporter has to convey a cargo across a membrane from a more or less neutral (cytoplasmic) into a more acidic (either vacuole, or apoplast) environment. It is well possible that the different charge of the nicotine cation at the two sides of the membrane is relevant for the directionality. Whether this transporter is located in the plasma membrane or in the tonoplast depends on the targeting of the vesicle flow in the trans-Golgi network (for review see Robinson and Pimpl 2014). There must also be an import transporter in the leaf that allows permeation of nicotine into the cytoplasm of leaf cells—the purine permease NUP1 (Kato et al. 2015) would qualify for this function. The subcellular localisation of these transporters has to be seen in the context of that for biosynthesis genes: while the key enzyme MPO1 (UniProt A4GZ88) is localised in the peroxisomes (Rajabi et al. 2017), consistent with a protein signature for import into the peroxisomal matrix (as predicted by https://wolfpsort.hgc.jp), the nicotine demethylase CYP82E4 (UniProt A1YJE3) harbours a transmembrane domain at amino acids 6–22, but lacks any canonical ER retention motif. Upon overexpression, the GFP fusion of CYP82E4 is dominantly labelling the ER (Rajabi et al. 2017). While the peroxisomes were long seen as autonomous organelles, it is now widely accepted that their membranes derive from the ER (Fig. 4, ①; for review see Tabak et al. 2003). Thus, the most likely scenario is that MPO1, which is imported into the peroxisomal matrix by virtue of its signal peptide (Fig. 4, ②), converts the *N*-methyl-Δ^1^-pyrrolinium cation into nicotine (Kato et al. 2007). The nicotine demethylase that is inserted by its *N*-terminal transmembrane domain into the ER membrane and protruding into the lumen will end up in the peroxisomal membrane protruding into the matrix of the peroxisome, where it now converts a part of the nicotine into nornicotine, while the remaining part of nicotine is exported into the cytoplasm (Fig. 4, ③). The very mild nsPEFs used in this experiment will only suffice to induce permeability of the plasma membrane, while the inner membranes remain tight. This assumption is supported by the finding that hardly any mortality was observed following the pulse treatment, which inevitably should be the case, once the integrity of the tonoplast would have been perturbed. The most straightforward explanation for the increased nicotine levels seen in the culture medium following the nsPEF treatment would be that the fraction of nicotine that is cytoplasmic (i.e. not protected by the tonoplast, nor the peroxisomal membrane) can leak out, while all the nornicotine which should be sequestered in the peroxisome, and later possibly also in the vacuole as consequence of membrane flow between peroxisome, Golgi, multivesicular body and tonoplast (Fig. 4, ④), and a large fraction of nicotine (around 2/3) remain sequestered. However, it cannot be totally excluded that nornicotine is secreted during an early phase of the pulse, since for technical reasons, a time interval of 2 h elapsed between pulsing and extraction.

## Conclusion

Intracellular sequestering of valuable plant compounds is a limiting factor in downstream processing, preventing continuous production cycles and necessitating batch processing. In the current work, we can demonstrate that a mild treatment with nanosecond pulsed electrical fields can release nicotine and anatabine into the culture medium without disturbing the viability of the cells. In contrast, nornicotine is not released. Thus, mild nsPEFs will lead to a fractionation of products dependent on their intracellular compartmentalisation. Although undesired in our model case, the anti-Alzheimer compound nornicotine, this electrofractionation might have biotechnological potential in other applications.

## Electronic supplementary material

Supplementary Figures S1 (a) High-performance liquid chromatography (HPLC) profile for the reference mixture of nicotineic alkaloids. b-f Diode array detection (HPLC-DAD; 260 nm) chromatogram of pure standards for (b) nornicotine, (c) anabasine, (d) anatabine, (e) anatalline (two isomeric forms), and (f) nicotine (from Rajabi et al. 2017) (DOCX 136 kb)

## Data Availability

All data generated or analysed during this study are included in this published article and its supplementary information files.

## Abbreviations

BY-2: *Nicotiana tabacum* L. cv Bright Yellow 2
HPLC: High-performance liquid chromatography
nsPEFs: nanosecond pulsed electric fields
*Ntab*MPO1: *Nicotiana tabacum N*-methylputrescine oxidase 1
*Ntom*CYP82E4: *Nicotiana tomentosiformis* cytochrome P_450_ (CYP82E4)
PI: Pulsing indices
WT: Wild type
