# Supplementary material for: Nanosecond pulsed electrical fields enhance product recovery in plant cell fermentation
Source: Protoplasma. 2020 Jul 10;257(6):1585–94. doi: 10.1007/s00709-020-01534-9 (PMC7567687; doi:10.1007/s00709-020-01534-9)

**Supplementary Figure S1 (a)** High-performance liquid chromatography (HPLC) profile for the reference mixture of nicotinic alkaloids. **b-f** Diode array detection (HPLC-DAD; 260 nm) chromatogram of pure standards for **(b)** nornicotine, **(c)** anabasine, **(d)** anatabine, **(e)** anatalline (two isomeric forms), and **(f)** nicotine (from Rajabi et al. 2017)


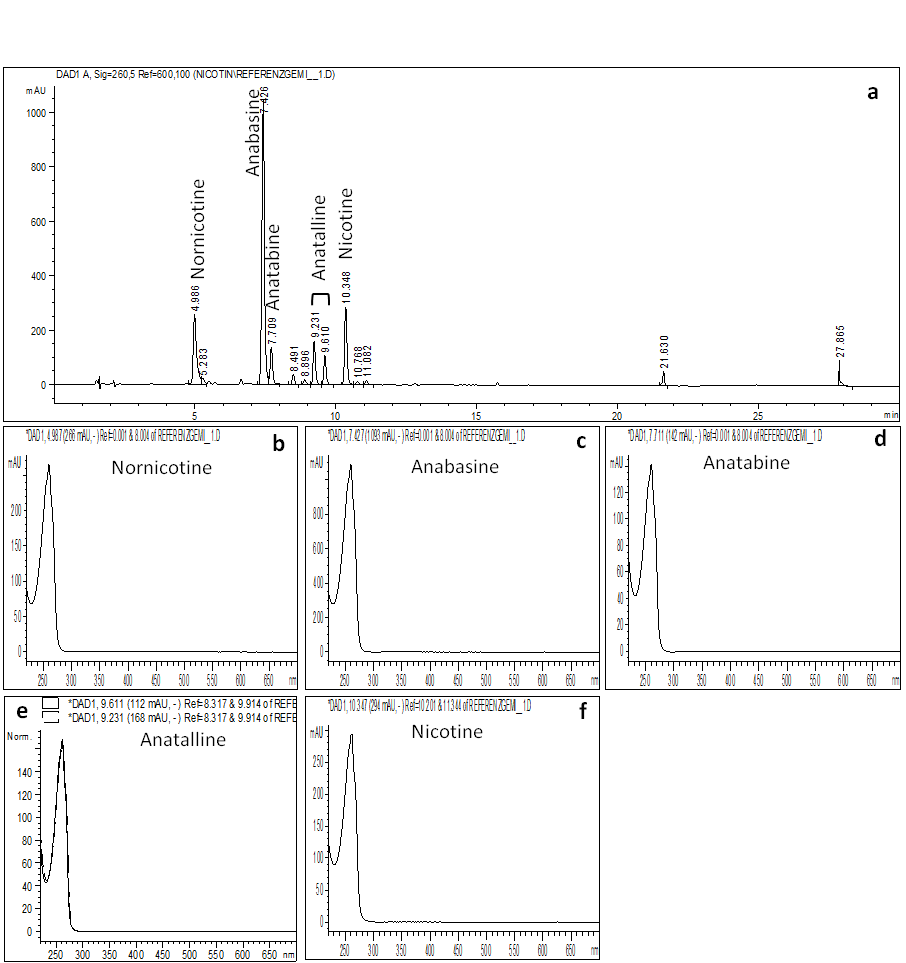

Supplement: Supplementary file 1 — (a) High-performance liquid chromatography (HPLC) profile for the reference mixture of nicotineic alkaloids. b-f Diode array detection (HPLC-DAD; 260 nm) chromatogram of pure standards for (b) nornicotine, (c) anabasine, (d) anatabine, (e) anatalline (two isomeric forms), and (f) nicotine (from Rajabi et al. 2017) (DOCX 136 kb) [file 709_2020_1534_MOESM1_ESM.docx]
